# Prolonged Treatment Time Deteriorates Positioning Accuracy for Stereotactic Radiosurgery

**DOI:** 10.1371/journal.pone.0123359

**Published:** 2015-04-20

**Authors:** Chun-Wei Wang, Yin-Chun Lin, Ham-Min Tseng, Furen Xiao, Chang-Mu Chen, Wei-Li Cheng, Szu-Huai Lu, Keng-Hsueh Lan, Wan-Yu Chen, Hsiang-Kuang Liang, Sung-Hsin Kuo

**Affiliations:** 1 Division of Radiation Oncology, Departments of Oncology, National Taiwan University Hospital, Taipei, Taiwan; 2 Department of Radiology, College of Medicine, National Taiwan University, Taipei, Taiwan; 3 CyberKnife Center, National Taiwan University Hospital, Taipei, Taiwan; 4 Division of Neurosurgery, Department of Surgery, National Taiwan University Hospital, Taipei, Taiwan; 5 Graduate Institute of Oncology, National Taiwan University, Taipei, Taiwan; University of Nebraska Medical Center, UNITED STATES

## Abstract

**Introduction:**

The accuracy of radiation delivery is increasingly important as radiotherapy technology continues to develop. The goal of this study was to evaluate intrafractional motion during intracranial radiosurgery and the relationship between motion change and treatment time.

**Methods and Materials:**

A total of 50 treatment records with 5988 images, all acquired during treatments with the CyberKnife Radiosurgery System, were retrospectively analyzed in this study. We measured translation and rotation motion including superior-inferior (SI), right-left (RL), anterior-posterior (AP), roll, tilt and yaw. All of the data was obtained during the first 45 minutes of treatment. The records were divided into 3 groups based on 15-min time intervals following the beginning of treatment: group A (0-15 min), group B (16-30 min) and group C (31-45 min). The mean deviations, systematic errors, random errors and margin for planning target volume (PTV) were calculated for each group.

**Results:**

The mean deviations were less than 0.1 mm in all three translation directions in the first 15 minutes. Greater motion occurred with longer treatment times, especially in the SI direction. For the 3D vector, a time-dependent change was observed, from 0.34 mm to 0.77 mm (*p*=0.01). There was no significant correlation between the treatment time and deviations in the AP, LR and rotation axes. Longer treatment times were associated with increases in systematic error, but not in random error. The estimated PTV margin for groups A, B and C were 0.86 / 1.14 / 1.31 mm, 0.75 / 1.12 / 1.20 mm, and 0.43 / 0.54 / 0.81 mm in the SI, RL, and AP directions, respectively.

**Conclusions:**

During intracranial radiosurgery, a consistent increase in the positioning deviation over time was observed, especially in the SI direction. If treatment time is greater than 15 minutes, we recommend increasing the PTV margins to ensure treatment precision.

## Introduction

A number of new radiotherapeutic techniques have been introduced into clinical use in recent years. All of these seek to make the dose distribution conformal as well as to reduce the dose on adjacent tissues as well as the possibility of adverse effects. Delivery accuracy during treatment is an important issue when using conformal techniques. To minimize the influence of set-up errors and organ motion, radiation precision needs to be improved to avoid dose insufficiency on the tumor or overdose to adjacent tissues.

For these reasons, new radiotherapy techniques are usually equipped with image guidance to analyze and reduce set-up errors for treatment. Most studies have focused on interfractional error during fractionation [1–6]. Relatively few, however, have discussed intrafractional error [7–11]. In these studies, the images were usually acquired before and after treatment to analyze displacements in patient positioning during treatment. Because of the relatively low sampling rates, the relationship between positioning error and treatment time has not been fully elucidated. This relationship is more important for intracranial tumors, which are often treated using single-fraction or hypofractionated radiotherapy with longer treatment time. Excessive and unexpected motion during the long treatment times not only reduces treatment effectiveness, but also increases the risk of complications.

To clarify this relationship, the CyberKnife Robotic Radiosurgery System (Accuray, Inc., Sunnyvale, CA, USA) can be used to increase the sampling frequency during treatment and to observe time-dependent changes in the positioning error of the head. The more accurate measurement enabled by this system could also provide a practical reference in optimizing the safe margin of the planning target volume (PTV) for clinical treatment.

## Methods and Materials

### Patients

Fifty patients who received cranial radiosurgery were enrolled in this study. These included 14 cases of meningioma, 19 cases of brain metastases, 8 cases of acoustic neuroma, 4 cases of arteriovenous malformation, 2 cases of pituitary tumor, one case of pineal tumor, one case of trigeminal neuroma, one case of and one case of hamartoma. The dose ranged from 12 to 30 Gy with a mean dose of 19.42 Gy. The number of fractions ranged from one to four.

### Treatment and image acquisition

**Fig 1:**
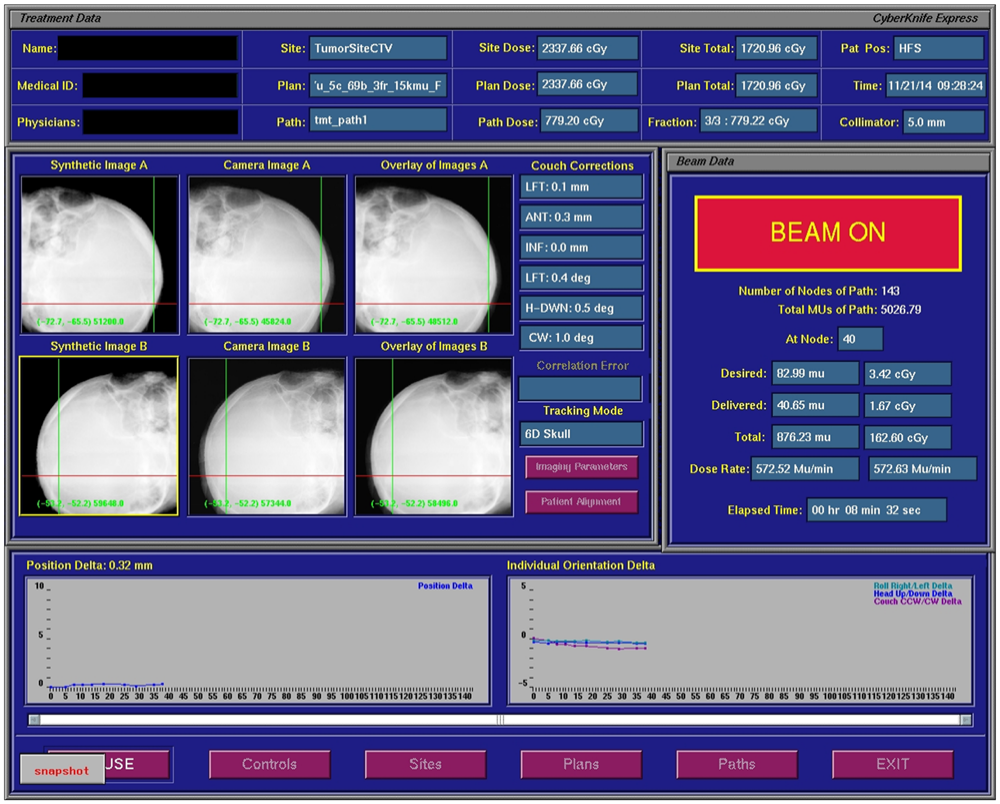
Image registration process. Comparison between the real time image and the DRR during radiosurgery.

The radiotherapy equipment used in the study was the CyberKnife Robotic Radiosurgery System with a 6MV photon beam. Each patient’s head was immobilized with a 2-mm thick thermoplastic mask, and then real-time images were obtained for image guidance during radiosurgery. Adjustments of either the patient position or the treatment couch were made until the positioning deviations were within the acceptable range and confirmed by the physicians. In this study, the image registration process used a 6D skull tracking system built into the CyberKnife (Fig 1). The system calculates the patient’s position to 0.1 mm and 0.1 degrees. The registration algorithm is designed in a multi-phase framework to achieve submillimeter tracking accuracy in real-time imaging [12]. The pixel number for the imaging detector is 1,024 × 1,024. The overall average CyberKnife system error is <0.95 mm root mean square. For treatment and image acquisition, the total number of beams in each treatment ranged from 103 to 311, with an average of 207. One real-time image was acquired every 1–3 beams to enable comparison with the digitally reconstructed radiograph (DRR). The robotic arms were automatically adjusted based on the analyzed positioning deviation to correct for set-up error. If the deviation of the translational axis was larger than 10 mm, the rotation angles of roll and tilt were larger than one degree, or the angle of yaw was larger than 3 degrees, the deviations were too large to be corrected by the robotic arm. In such cases, the technicians would enter the treatment room to adjust the patient position or treatment couch. Once this was completed, the recorded values for the deviations were reset, rather than adopting the original values from the patients. To accurately illustrate the trend in deviation changes during treatment, we analyzed only the data obtained after treatment until the treatment couch was adjusted or the patient’s position was altered.

The image detector consisted of scintillator screens composed of cesium iodide (CsI). The image size was 1024x1024 pixels and the receiving area was 40x40 cm^2^. Real-time imaging was centered with the orthogonal X-ray images. The image acquisition parameters were approximately 100 kV, 100 mA and 100 ms. The real-time images were compared with the DRR from the simulation CT images to measure deviation in each translation and rotation axis. The average treatment time was 57.4 min (range: 45.6–83.7 min). The time to acquire images ranged from 19.4 to 72.8 sec, with an average of 30 sec. A total of 5,988 images were obtained, with approximately 120 images in each treatment record.

### Definition of axes

The coordinate system used in this study was based on the patient’s position while lying on the couch. The X-axis was in the superior-inferior (SI) direction; the direction toward the feet was positive and head was negative. The Y-axis was in the right-left (RL) direction; the direction toward the left was positive and right was negative. The Z-axis was in the anterior-posterior (AP) direction; the direction toward the anterior was positive and posterior was negative. The rotation axis was defined as positive if rolling to the right for the X-axis, and as negative if rolling to the left. It was defined as positive if the head was tilted upward for the Y-axis, and as negative if the feet were tilted upward. For the Z-axis, yawing counter-clockwise was positive and yawing clockwise was negative.

### Data analysis

Of the 50 patients enrolled in this study, 33 did not require adjustment of the position or couch during the treatment. Another 17 cases were recorded from the beginning to the first adjustment of the treatment couch or patient position to ensure that the recorded values were based on the same baseline. The deviations of the 6 axes (X, Y, Z, roll, tilt and yaw) were recorded at each sampling point and the deviation in 3D vectors was obtained by calculating deviations in the three translational axes. The calculation formula was as follows:

$$3D=\sqrt{SI^{2}+AP^{2}+RL^{2}}.$$

The observation time was typically no longer than 45 min because after that time, in most cases, the couch or the patients’ position would need to be adjusted. As noted above, the values recorded after such adjustment would be biased and were not included in the analysis. To demonstrate the length of treatment and the trend in position changes, we analyzed the deviation in positioning using the images from the beginning of the treatment as the baseline and recording the changes in position at different time points.

To estimate an adequate margin for PTV, we used the definition from van Herk et al. [13]. Systematic (Σ) and random errors (σ) were calculated using the method proposed by Bijhold et al. [14]. The safe margin for PTV would be the sum of 2.5 times the systematic errors and 0.7 times the random errors (M = 2.5Σ+0.7σ). To illustrate time-dependent changes in deviations and an adequate margin for PTV, the treatment time was divided from the beginning (0–45 min) into three sessions using 15-min intervals: group A: 0–15 min; group B: 16–30 min, and group C: 31–45 min. Statistical calculations were performed using SPSS software, version 16 (SPSS Inc., Chicago, IL). All reported p values were two-tailed and considered to be significant in the presence of a p<0.05

### Ethics statement

The study protocol was approved by the Research Ethical Committee of National Taiwan University Hospital (NTUH-201406060RIC). The patients' medical data were anonymized prior to access and analysis. The institutional review board has waived the need for written informed consent from study subjects because all potentially patient-identifying information was removed prior to data analysis.

## Results

**Fig 2:**
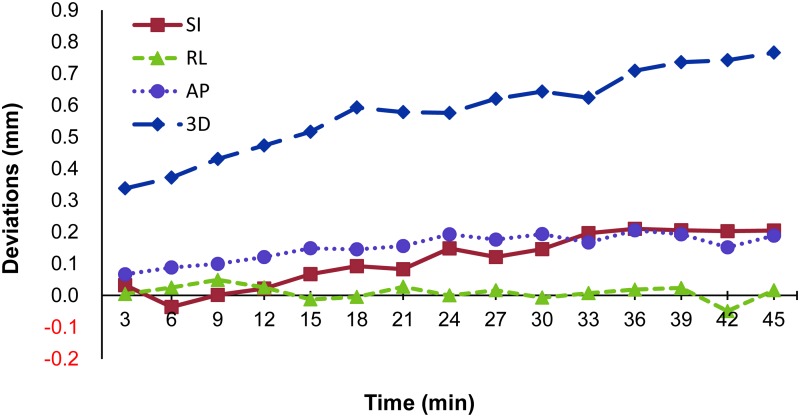
Translational deviations. Intrafractional motion in the translational axes during treatment.

**Fig 3:**
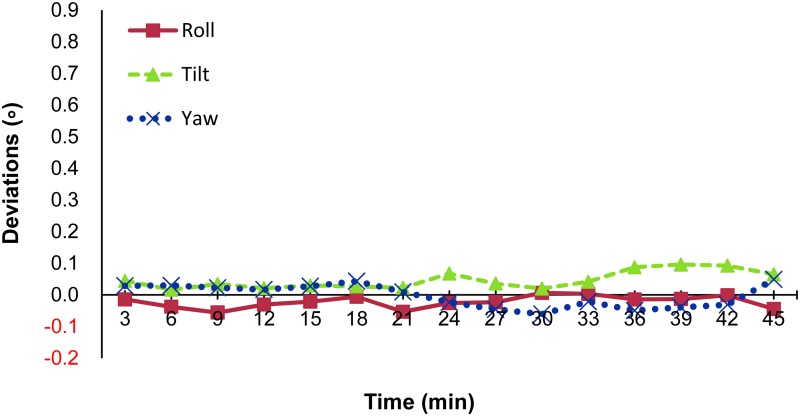
Rotation deviations. Intrafractional motion in the rotation axes during treatment.

Values were analyzed every 3 min from 0 to 45 min, with a total of 15 values for each treatment record (Figs 2 and 3). The SI deviation increased over time from 0.03 mm to 0.20 mm (*p* = 0.02) while the AP deviation increased from 0.07 mm to 0.19 mm (*p* = 0.07). The RL direction did not deviate significantly: roughly 0.01 mm (*p* = 0.15). For the 3D vector, a time-dependent change became evident. The 3D deviation more than doubled, from 0.34 mm to 0.77 mm (*p* = 0.01). All of the rotation axes, including roll, tilt and yaw, remained within 0.1 degrees during the treatment. As shown in Fig 3, there was no significant association between rotation axes and treatment time (*p*>0.05).

**Fig 4:**
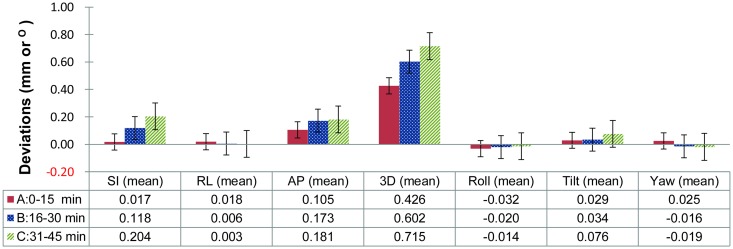
Deviations among the different time intervals. Mean deviations in different 15-min sessions during treatment: group A: 0–15 min from treatment beginning; group B: 16–30 min; and group C: 31–45 min.

To understand whether deviations in the patients’ positions changed with time, the mean deviation values were compared among the different time intervals 0–15 min, 16–30 min, and 31–45 min (Fig 4). With the image-guided calibration before treatment, the mean deviations of the three translational axes were less than 0.1 mm in the first 15 min. The changes in the three rotation axes were all within 0.1 degrees. However, the mean deviations in the SI direction increased significantly after 15 min, from 0.02 mm to 0.20 mm (*p* = 0.003). Though there was no significant change in the AP (*p* = 0.09) or RL (*p* = 0.83) directions before and after the first 15 min, the 3D vector increased statistically significantly from 0.43 mm (0–15 min) to 0.60 mm (16–30 min, *p*<0.001). The deviation in the 3D vector still increased consistently after 30 min, from 0.60 mm to 0.72 mm (*p* = 0.004). For the rotation axes (roll, tilt and yaw), there was no statistically significant difference in the three 15-min sessions during the treatment (*p*>0.05).

With this study, we analyzed the measured data and estimated the adequate PTV margins. The systematic errors, random errors and adequate margins of each translational axis in the three 15-min sessions are listed in Table 1. With the systematic errors, consistent increases over time were observed for all three translation axes. There was no clear time-dependent change for the random errors. With the initial calibration before treatment, using an image-guided tool, the PTV margins of the three translational axes were less than 1 mm for the first 15 min of treatment. Larger PTV margins (>1mm) would be required, though, if the treatment time were more than 15 min, especially in the SI and RL directions.

**Table 1:** The systematic errors (Σ), random errors (σ) and estimated PTV margins (M) in the three 15-min sessions.

	X (superior-inferior)			Y (right-left)			Z (anterior-posterior)		
Time sessions	Σ (mm)	σ (mm)	M (mm)	Σ (mm)	σ (mm)	M (mm)	Σ (mm)	σ (mm)	M (mm)
0~15 min	0.27	0.25	0.86	0.25	0.18	0.75	0.14	0.11	0.43
16~30 min	0.40	0.21	1.14	0.40	0.18	1.12	0.18	0.14	0.54
31~45 min	0.46	0.22	1.31	0.43	0.17	1.20	0.28	0.15	0.81

*Abbreviations*: Σ = systematic errors, σ = random errors, and M = estimated PTV margins

**Fig 5:**
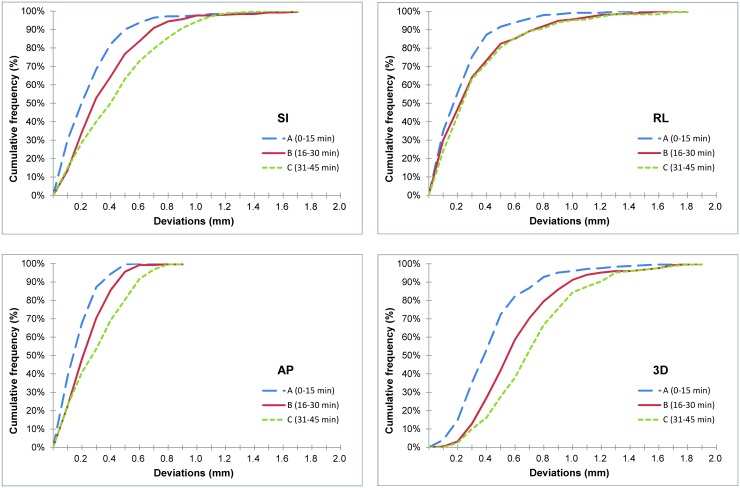
Cumulative frequency of deviations. The cumulative frequency greater than or equal to the value in the corresponding deviations for the translational axes and the 3D vector.

Fig 5 shows the cumulative frequency greater than or equal to the value in the corresponding deviations for the translational axes and the 3D vector; the values of deviation were analyzed in absolute values. To cover the 95% probability of deviations occurring, the margins required during the first 15 min for the SI, RL and AP directions were 0.65 mm, 0.65 mm and 0.41 mm, respectively. Between 16 and 30 min, the required margins were 0.85 mm, 0.93 mm and 0.49 mm for 95% coverage in the SI, RL and AP axes, respectively. For treatment beyond 30 min, the adequate margins would be 1.02 mm, 0.98 mm and 0.67 mm, respectively. In this actual implementation, if the treatment time was less than 15 min, the 1-mm safe margin could cover more than 95% of the deviations for all translational axes. Between 16 and 30 min, 2.4% and 4.4% of the patients showed deviations more than 1 mm in the SI and RL directions, respectively. After 30 min, these numbers were 5.6% and 4.8%, respectively. For the 3D vector, we observed deviations more than 1 mm in 4.0% of the patients during the first 15 min, in 8.8% of the patients between 16 and 30 min, and in 15.6% of the patients between 31 and 45 min.

## Discussion

For head and neck irradiation, most studies focus on the interfractional (day-to-day) change. The intrafractional change during treatment has been less widely addressed. For routinely fractionated radiotherapy, the intrafractional change might be small as the treatment time is usually less than 10 min. For single or hypo-fractionated radiosurgery, however, the treatment time will increase remarkably due to the large prescription dose used. For this reason, consistency of patient positioning is an important issue. In the present study, we observed that deviations in the translational axes increased over time. This result was similar to that reported by Hoogeman *et al*. [11], who recorded time intervals of 15 min and showed that deviations increased over time. The systematic error increased nearly linearly with time to 0.5, 0.5 and 0.3 mm (at 15min) in the SI, LR and AP directions, respectively. In our work, we took measurements for up to 45 min and noted a gradual increase in the deviation in the 3D vector. Between those with meningioma (14 cases) and brain metastases (19 cases), we could not find any statistically difference (S1 Fig, p = 0.1). In the patients enrolled in this study, the tumor locations were different even with the same diagnosis. This may explain why the deviation change was not significantly disease-specific in this study. The increase in 3D deviation mainly came from the SI direction. During the 45 min of treatment, deviations in the RL direction remained within 0.1 mm, the lowest values among the translational axes. For the rotation axes, the tilt axes exhibit a trend of greater deviation when the treatment time increased (Fig 3). This may explain the deviation in the AP and SI directions, which gradually increased over time (Fig 2). Even with immobilization using the thermoplastic mask, the tilt of the head and jaw may have a greater positioning variation over a longer treatment time. This exhibition may contribute to the deviations in the SI and AP directions.

There are several methods with which to quantify deviations in position. Some studies have investigated the intrafractional movement detecting the changes using the infrared reflection from reflective material fixed on the head [7, 9, 15]. The disadvantage of this method is that the data recorded may vary based on the amount and the fixation positions of reflective material used. For patients treated in the supine position, the reflective material can only be attached to the front or bilateral sides of the head, not to the posterior parts. Therefore, only deviations of anterior part of the head can be obtained using this system with infrared detection. Another limitation found in most published studies is the sampling frequency and the relatively low numbers of patient enrolled. The commonly used method is to acquire planar images before and after treatment and, using software, compare the changes in position between the two time points [7, 8]. Thus, only overall deviations between two sets of images can be determined, and time-dependent changes during treatment are not revealed. To address these limitations, the present work used the CyberKnife with increased sampling frequency to evaluate deviations in position and the relationship between deviations and treatment time. We also enrolled more patients for analysis—a total of 50 cases—to accurately quantify the deviations during the intracranial radiosurgery.

In previously reported studies, the intrafractional motion of the translational axes was mostly between 0.1 mm and 2.0 mm for the head and neck region [8, 9, 11, 16]. The values in our study were lower than in the published data. The mean deviations during treatment (0–45 min) were 0.11, 0.15 and 0.01 mm in the SI, AP, and LR directions, respectively. Although the deviations increased over time, the mean deviations in the third 15-min session (31–45min) were still within 0.2 mm in the three translational directions (Fig 4). Several factors might have contributed to the lower values in the present study. First, following the recommendations of the manufacturer of the CyberKnife, the deviation was initially corrected and minimized to within 1 mm or 1° before the treatment began. This initial calibration might have helped to minimize the values recorded during the treatment. Second, the difference in regions-of-interest for image registration and analysis may contribute to the variation in measured data. In our study, positioning deviations were analyzed by the software (6D skull) built into the CyberKnife. The cranial bony structures were the main targets for image comparison; the neck and the spine were not included, as they have been in other studies [8, 17]. Generally, the mandibular bone and the spine exhibit greater variation than the cranial bone. If these movable joints were included in the analysis, the deviations recorded would be larger. Third, differences of measurement tools and sampling frequency might contribute to the lower values. As mentioned above, some studies have used infrared reflective materials to measure deviations during treatment [7, 9, 15]. The reflective materials were mostly located in the front side of the head and neck; the data collected only represents positioning changes close to the reflective materials, not those of whole cranium. Such differences might also contribute to the discrepancy in results.

The margins for PTV are generally determined by the treatment location and the types of equipment used. Little attention has been paid to the influence of position deviations related to the length of the treatment. To offer a guide for clinical practice, in this study we sought to determine the adequate margins for PTV. According to the formula presented by van Herk *et al*., systematic errors and random errors must be obtained when calculating PTV margins [13]. While random errors did not change significantly, systematic errors increased consistently over time (Table 1). Compared to the values in the first 15 min, the systematic errors in the 16–30 min session increased by 45.0%, 58.1% and 27.6% for the SI, RL and AP axes, respectively. In the 31–45 min session, the deviations increased by 69.1%, 70.9% and 104.4%, respectively. Systematic errors played a greater role than random errors in calculating the margins of PTV (M = 2.5Σ+0.7σ) [13]. From our analyzed data, it can be inferred that the deviations were within 1 mm if the treatment time was less than 15 min. However, at least 1-mm margins for PTV might be considered if the treatment time is greater than 15 min, even with initial calibration before treatment begins. Another analysis in this study yielded a similar finding (Fig 4). As noted above, 4.0% of the patients exhibited deviations of more than 1 mm in the 3D vector. For treatment beyond 15 min, 8.8% of the patients showed deviations of more than 1 mm.

Although van Herk’s formula is one of those that has been widely used to estimate the PTV margin, it is derived from probability calculations based on multi-fraction treatments only. For single-fraction radiosurgery treated by a specific machine, Zhang *et al*. illustrated an innovative margin formula that is machine-specific and accounts for a nonzero mean systematic error, and it may be more suitable for single-fraction radiosurgery in clinical applications [18]. In the present study, we demonstrated an evidently time-dependent change in deviations during intracranial radiosurgery. Based on this observation, the adequate margin for patients with a longer treatment time may need to be adjusted depending on the fractionation, machines, and clinical judgment.

## Conclusions

For stereotactic radiosurgery, a constant concern is that the treatment accuracy may be compromised by uncertainties in the patient's position. Prolonged radiosurgery treatment times can affect patient comfort. Our study demonstrated that during intracranial radiosurgery, a consistent increase in the positioning deviation over time was observed, especially in the SI direction. With high-precision equipment and image guidance, intrafractional motion is within 1 mm if the treatment time is less than 15 minutes. If image-guided calibration is not performed and the treatment time is greater than 15 minutes, we recommend increasing the PTV margins to ensure precision of treatment for intracranial radiotherapy. These findings will enhance stereotactic set-up precision.

## Supporting Information

S1 Fig 3D deviations between brain metastases and meningioma. (TIF)
